# In-situ twistable bilayer graphene

**DOI:** 10.1038/s41598-021-04030-z

**Published:** 2022-01-07

**Authors:** Cheng Hu, Tongyao Wu, Xinyue Huang, Yulong Dong, Jiajun Chen, Zhichun Zhang, Bosai Lyu, Saiqun Ma, Kenji Watanabe, Takashi Taniguchi, Guibai Xie, Xiaojun Li, Qi Liang, Zhiwen Shi

**Affiliations:** 1 Key Laboratory of Artificial Structures and Quantum Control (Ministry of Education), Shenyang National Laboratory for Materials Science, School of Physics and Astronomy, Shanghai Jiao Tong University, Shanghai, 200240 China; 2 Research Center for Functional Materials, National Institute for Materials Science, 1-1 Namiki, Tsukuba, 305-0044 Japan; 3 International Center for Materials Nanoarchitectonics, National Institute for Materials Science, 1-1 Namiki, Tsukuba, 305-0044 Japan; 4 National Key Laboratory of Science and Technology on Space Microwave, China Academy of Space Technology (Xi’an), Xi’an, China

**Keywords:** Mechanical and structural properties and devices, Nanophotonics and plasmonics

## Abstract

The electrical and optical properties of twisted bilayer graphene (tBLG) depend sensitively on the twist angle. To study the angle dependent properties of the tBLG, currently it is required fabrication of a large number of samples with systematically varied twist angles. Here, we demonstrate the construction of in-situ twistable bilayer graphene, in which the twist angle of the two graphene monolayers can be in-situ tuned continuously in a large range with high precision. The controlled tuning of the twist angle is confirmed by a combination of real-space and spectroscopic characterizations, including atomic force microscopy (AFM) identification of crystal lattice orientation, scanning near-field optical microscopy (SNOM) imaging of superlattice domain walls, and resonant Raman spectroscopy of the largely enhanced G-mode. The developed in-situ twistable homostructure devices enable systematic investigation of the twist angle effects in a single device, thus could largely advance the research of twistronics.

## Introduction

Two-dimensional (2D) materials and the effects of their interlayer van der Waals (vdW) interaction are topics that attracted unprecedented attentions. With the development of the transfer technique, these atomically thin materials can be stacked layer by layer like the Lego blocks^1^. Due to the subtle shift of atom position and the delicate reconstruction of the band structure, a slight variation in the twist angle of stacking may even result in an astonishing change in the electrical and optical properties^2–9^. For example, electrically, only at the magic angle (~ 1.1°), the twisted bilayer graphene (tBLG) experiences flat electronic bands and exhibits strong correlation phenomena, such as Mott insulator^10^, superconductivity^11^, topological Chern insulator^12^ and ferromagnetism^13^. Optically, the resonant Raman^14,15^ and resonant absorption^16,17^ of the tBLG require the energy difference of the two van Hove singularities in the valence and conduction bands to match the excitation photon energy, thus can only occur for specific twist angle.

The most commonly used method to fabricate a twist device is the “tear-and-stack” technique^18,19^. The shortcoming of this method is that the twist angle of devices achieved this way are not tunable any more after the device fabrication. As a result, when studying twist angle dependent properties, one has to collect data from a series of devices with different twist angles. However, when comparing data measured in different devices, it is generally hard to rule out irrelevant factors and look into the pure twist angle effect. Therefore, an in-situ dynamically rotatable device is highly desirable. In light of this, some rotatable-device-fabrication methods have been developed. One is the thermal rotation method^20,21^, where a monolayer material on the atomically flat surface can drift and rotate to a steady state in a high-temperature annealing process. However, the thermal rotation is of less control. This issue can be solved by a scanning tunneling microscope (STM) folding technique^22^, where a precisely controlled STM tip is used to pick up a graphene edge and then fold it to form tBLG. Unfortunately, the rather small sample size (of only ~ 100 nm) obtained this technique is disadvantageous for further optical or electrical measurements. This problem was resolved by using an atomic force microscopy (AFM) manipulation technique, where an AFM tip is used to push a micrometer-sized thick hBN gear wheel on top of a graphene monolayer to construct twistable graphene/hBN heterostructures^23,24^. The success in building such large-sized heterostructures is beneficial from two key factors. First, the thick hBN is quite rigid, which allows the manipulation of the whole structure by using a tiny AFM tip. Second, the graphene-hBN interface is superlubricity, therefore it is feasible to overcome the interfacial friction^25,26^. However, this method can not be directly migrated to construct in-situ tunable tBLG due to the difficulties in manipulating a large-area flexible graphene sheet with a tiny AFM tip. Up to now, in-situ manipulation of the rotational angle in large area tBLG homostructure is still not available.

Here, we report a technique for constructing twistable homostructure devices, and show an example that the twist angle of a bilayer graphene device can be in-situ manipulated continuously. The device contains a large piece of bottom-layer graphene, a relatively smaller piece of top-layer graphene and an hBN hollow gear in between. In the central hollow area of the gear, the top-layer graphene gets in touch with the bottom-layer graphene, forming a region of tBLG. The gear together with the top-layer graphene can be in-situ rotated by an AFM tip, thus the twist angle of the central region of tBLG can change accordingly. The change of the twist angle is revealed by both the AFM identification of crystal lattice direction and the scanning near-field optical microscopy (SNOM) imaging of the superlattice domain wall. Raman spectrum are also carried out to in-situ measure the twist angle dependence of the device. The constructed bilayer graphene devices with in-situ dynamical tunability offer opportunities to investigate the twist angle effects in a single device.

## Results

### Structure of the twistable bilayer graphene device

**Figure 1:**
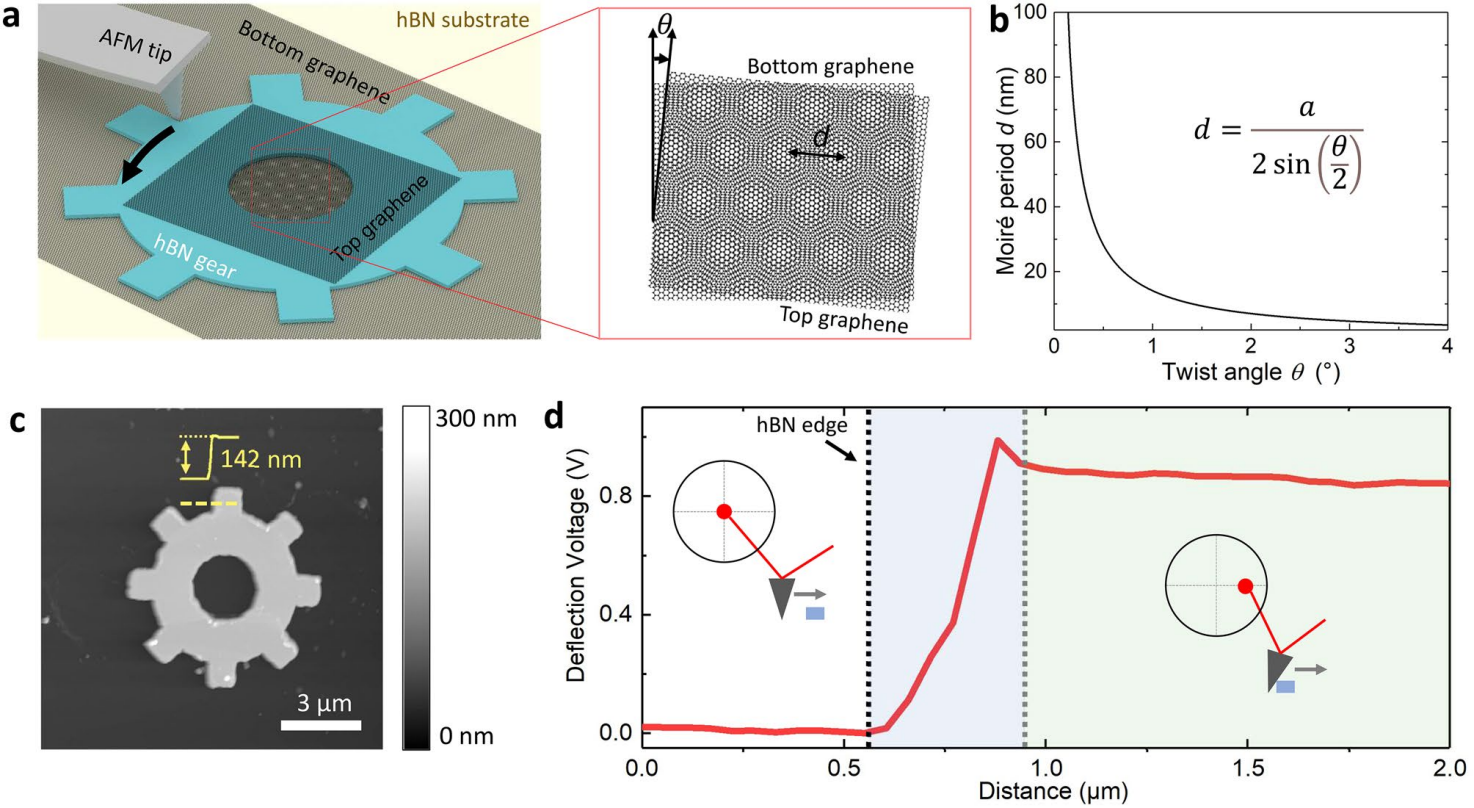
A twistable bilayer graphene device. (**a**) Schematic diagram of the structure of an angle-tunable twisted bilayer graphene (tBLG) device. It consists of the two graphene monolayers separated by a hBN gear on another hBN substrate. Moiré superlattice of period *d* is formed in the overlapping area in the gear center. (**b**) The relation between the twist angle *θ* and the Moiré period *d*. (**c**) The topography of a twistable device. (**d**) The tip deflection versus tip moving distance during the pushing processes of the hBN gear.

Figure 1a shows the schematic of our device structure. The device consists of a large-piece of bottom-layer graphene, a small-piece of top-layer graphene, and a preshaped hBN gear in between. The whole device is supported by another large piece of hBN flake on the SiO_2_/Si substrate (see more details in Methods Section and Supplementary Information). The bottom hBN flake is aimed at offering an atomically flat platform to avoid the surface fluctuation of the SiO_2_/Si substrate which could hinder the movement of the hBN gear. The central hole of the hBN gear is designed to provide an overlap area of two graphene monolayers to form the tBLG area. Because of the superlubricity effect between the interlayers of vdW materials, the friction between the hBN gear and the bottom graphene layer is low enough so that the hBN gear is rotatable through AFM tip pushing^27^. The top-layer graphene, with its main part sitting on the hBN gear, will follow the motion of the hBN gear. In this way, the twist angle *θ* of the central bilayer graphene region can be controlled by rotating the hollow hBN gear with an AFM tip. Different twist angle *θ* leads to moiré patterns of different periods *d*, as:

1 $$d=\frac{a}{2\mathrm{sin}\left(\theta /2\right)}$$

where *a* = 0.246 nm is the lattice constant of graphene. This relation at near zero-twist angle is explicitly shown in Fig. 1b. The moiré superlattice period is divergent as the twist angle *θ* reduces to zero. This is very different from a typical twisted heterostructure consisting of different crystals, where the maximum period is typically finite. For instance, the maximum superlattice period of a graphene/hBN heterostructure is around 15 nm^28^. This feature gives homostructure a capability of band control in a larger range.

**Figure 2:**
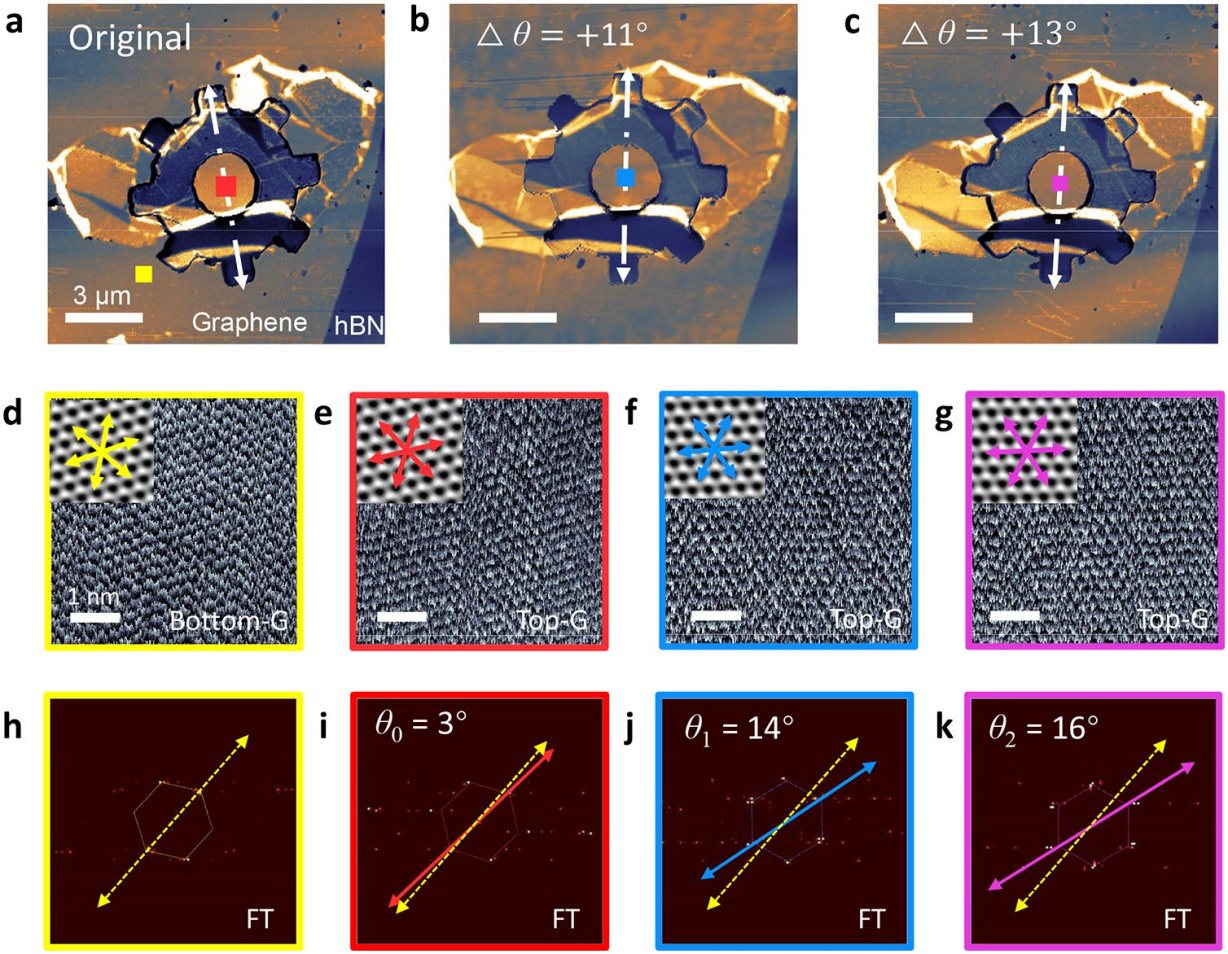
Manipulation of the twist angle. (**a**–**c**) Near-field infrared imaging of the same device at different twist angles. Δ*θ* indicate the angle change of the hBN gear relative to its original orientation. Scale bar: 3 μm. The dashed line arrows indicate a representative direction of the hBN gear. (**d**) High-resolution AFM image of the bottom layer graphene. Scale bar: 1 nm. The arrows indicate the zigzag direction of the graphene lattice. (**e–g**) High-resolution AFM images of the top layer graphene in the colored square regions in (**a**–**c**). (**h**–**k**) The 2D Fourier transform of figures (**d**–**g**). The twist angle *θ* between the bottom (yellow) and the top (others) layer graphene are 3°, 14°, 16°, respectively.

Figure 1c shows the topography of a device scanned by tapping-mode AFM. The bright area indicates the shape of an hBN gear with thickness ~ 140 nm, ensuring its rigidity. On the contrary, the graphene monolayer is only 0.34 nm, and therefore is hard to see from the topography image. Thanks to the strong infrared response of the Dirac electrons in graphene, an infrared SNOM system with a laser beam at 10.6 μm can easily identify the graphene layers in our device (Fig. 2a–c).

### Procedure of AFM-tip manipulation of the twist angle

We now introduce how to rotate the top-layer graphene using an AFM tip. The AFM tip is to exert a planar force on the hBN gear’s protrusions to rotate the hBN gear and the top-layer graphene. To ensure this works, two conditions need to be fulfilled. First, the hBN gear can move and rotate relative to the underneath bottom-layer graphene. This is achievable due to the ultra-low friction between the hBN and graphene. Note that the bottom layer graphene should be large enough so that it can stick to the bottom hBN substrate and not rotate together with the hBN gear. Second, the top layer graphene should rotate together with the hBN gear. This can be achieved by considering two aspects: (1) increase the top-surface roughness of the hBN gear. Luckily, after the lithography and etching process (over etched), the surface of the hBN gear is quite rough, which favors the top graphene to follow exactly the movement of hBN gear. (2) Choose a small size top-layer graphene sheet to reduce the graphene-graphene contact area outside the hBN gear.

We use the lift-mode of the AFM to realize the rotation, where the tip first line-scans the protrusion and then pushes the protrusion by rescanning the line. During the second scan, the tip lifts down by ~ 1 μm to ensure the tip keeps contacting with the hBN gear throughout the pushing process. The AFM tip deflection signal in this process is recorded in Fig. 1d. Three different regimes of the pushing are identified: (1) As the tip lifts down and drags along the bottom graphene surface, tip-substrate friction results in a small and steady-state tip deflection. We refer to this as the substrate friction regime. (2) When the tip encounters the edge of a gear protrusion, sudden increase of mechanical resistance induces a torque to the AFM tip as well as an increase of tip deflection signal. If the tip pushing force is smaller than the maximum static friction force, the hBN gear will keep standing still. We refer to this stage as static friction regime. (3) Once the hBN gear starts to move, the friction force between the top structure and bottom graphene layer reduces from the maximum static friction to the kinetic friction, which causes the tip deflection to relax down slightly. We refer to this stage as kinetic friction regime. The distance that the AFM tip moves in the kinetic friction regime corresponds to the exact motion of the hBN gear.

To confirm the rotation of the hBN gear and the top-layer graphene, we carried out SNOM and high-resolution AFM measurements before and after each pushing process. Near-field infrared images of three states of the hBN gear is shown in Fig. 2a–c. The dashed line arrows indicate a representative direction of the hBN gear, from which one can clearly see the rotation of the hBN gear relative to the stationary bottom-layer graphene. The rotation angles Δ*θ* of the hBN gear are measured to be 11° and 13°, respectively.

Furthermore, we are able to obtain the lattice orientation of both the bottom- and the top-layer graphene through high-resolution lateral force AFM, from which the twist angle can be directly obtained. We first scan the lattice orientation of the bottom-layer graphene. The scanning position is marked with a yellow square in Fig. 2a, and the scanned lattice image is shown in Fig. 2d. To see the lattice orientation more clearly, we performed Fourier transformation (FT) of the lattice image, as shown in Fig. 2h, in which the yellow arrow indicates one of the zigzag directions in the reciprocal space. The FT filtered clean lattice is displayed as an inset of Fig. 2d, and the three yellow arrows indicate the zigzag directions of the bottom layer graphene. The lattice images of the top-layer graphene in the center of the gear and their FTs are shown in Fig. 2e–g, i–k. Thus, the twist angle *θ* for the three states are extracted to be 3°, 14°, 16°, respectively, from Fig. 2i–k.

The changes of twist angle are 11° and 13° respectively, after the two pushing manipulations, matching exactly the rotation angle of the hBN gear, in the high-resolution AFM image. The perfect match between the gear rotation angles and the change of twist angle reflects that the rotation of the top-layer graphene follows exactly the rotation of the hBN gear. This is crucial to the twist angle control and beneficial to its further applications. In the following two experiments, we demonstrate the application of both large-range tuning and small-range fine-tuning of the twist angle in finding resonant Raman and control of moiré superlattice period.

### Large-range tuning of the twist angle to find the critical angle for resonant Raman

**Figure 3:**
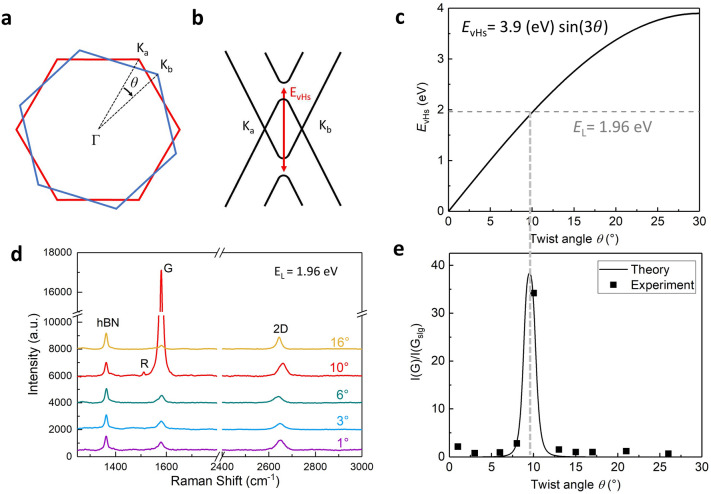
Large-range tuning of the twist angle to find the critical angle for resonant Raman. (**a**) The first Brillouin zone (BZ) of twisted bilayer graphene misoriented by *θ*. (**b**) Electronic bands in the vicinities of two Dirac cones. Van Hove singularities (vHs) are induced by band overlap. (**c**) Energy difference of the two vHs versus the twist angle *θ.* The gray dashed line indicates the excitation laser energy *E*_L_ = 1.96 eV in our Raman measurement, corresponding to a twist angle of ~ 10°. (**d**) Raman spectra of a twistable bilayer graphene devices at a few representative twist angles. At *θ* = 10°, the graphene G-peak is largely enhanced. (**e**) The normalized graphene G-peak intensity as a function of the twist angle *θ*. Large enhancement at *θ* = 10° corresponds to the predicted resonant Raman.

The AFM-tip manipulation of the hBN gear allows tuning of the twist angle in a large range without limitation, which can be used to find the critical angles for the transitions of some physical properties. Thanks to the micrometer-scale size, our device is applicable to far-field optical measurement. As an example, we demonstrate large-range tuning of the twist angle to find the critical angle for resonant Raman. Recent theoretical and experimental works have reported the presence of van Hove singularities (vHs) in the density of π electron states in tBLG. These vHs induce very strong enhancement in the Raman G band intensity. The enhancement only occurs at specific twist angle *θ* for a given incident laser energy *E*_L_. For two graphene layers misoriented by angle *θ*, the Brillouin zones of the two layers also rotationally shift by *θ*, as shown in Fig. 3a. Band-structure modifies most where the Dirac cones of top and bottom layers crossed. Figure 3b shows the simplified electronic band-structure in the vicinity of the cross of these two Dirac cones. In this region, the density of states (DOS) of double-layer graphene exhibits vHs. The energy difference between conduction and valence vHs scales with the rotational angle, *E*_vHs_ = 3.9(eV)sin(3*θ*), as shown in Fig. 3c. The resonant Raman occurs when excitation energy *E*_L_ = *E*_vHs_. In our Raman measurement, the excitation energy *E*_L_ = 1.96 eV (633 nm in wavelength), which requires a twist angle around 10° for the resonant Raman.

With a twistable bilayer graphene device introduced above, we in-situ tune its twist angle in a large range from 0 to 30 degrees, and record its Raman spectra. Figure 3d shows Raman spectra at a few representative twist angles, 1°, 3°, 6°, 10° and 16°. In the spectra, three main Raman peaks can be observed. The left peak around 1370 cm^−1^ is the hBN in-plane phonon peak, which is largely constant and therefore can be used to normalize the Raman intensity of graphene with different twist angle. The right broad peak around 2650 cm^−1^ is the 2D peak of the tBLG. The peak around 1580 cm^−1^ is the G peak of tBLG. The intensity of the G peak is significantly enhanced when the twist angle is around 10° as expected from resonant Raman condition *E*_L_ = *E*_vHs_. The quantitative enhancement factor of the G peak of the tBLG relative to the single layer graphene can be described using a second order time-dependent perturbation theory^29^, as $\frac{I\left({G}_{tBLG}\right)}{I\left({G}_{single}\right)}={\left|\frac{M}{\left({E}_{L}-{E}_{vHs}-i\gamma \right)\left({E}_{L}-{E}_{vHs}-\hslash {\omega }_{G}-i\gamma \right)}\right|}^{2}$, where ℏω_G_ is the energy of the G phonon, *M* is the product of the matrix elements for electron-photon and electron–phonon interactions, and *γ* is the resonance window width. This equation can well describe the G peak intensity observed experimentally for different twist angles, as shown in Fig. 3e. Other twist angle dependent Raman parameters, such as the peak shift and full width half maximum (FWHM), can be found in Fig. S3.

The advantage of our technique is that we can in-situ tune the twist angle and obtain twist angle dependence in a single device, which can largely reduce the amount of experimental workload. In addition, we can exclude the influence from different samples and substrate factors and obtain pure twist angle dependent information. The demonstrated method can also be used for investigation of other twist angle dependent optical properties, such as photoluminescence, second harmonic generation, photocurrent, and circular dichroism.

### Small-range fine-tuning of the twist angle to control the moiré superlattice period

Our in-situ twist technique not only exhibits tuning ability in a wide range, but also shows unprecedented high precision. Since the moiré period of tBLG diverges at zero twist angle, the period is extremely sensitive to the twist angle near the zero twist. Therefore, the control of the moiré period requires precise tuning of the tiny twist angle near zero. Here, we demonstrate control of the moiré period of tBLG using a combination of AFM and SNOM characterizations.

**Figure 4:**
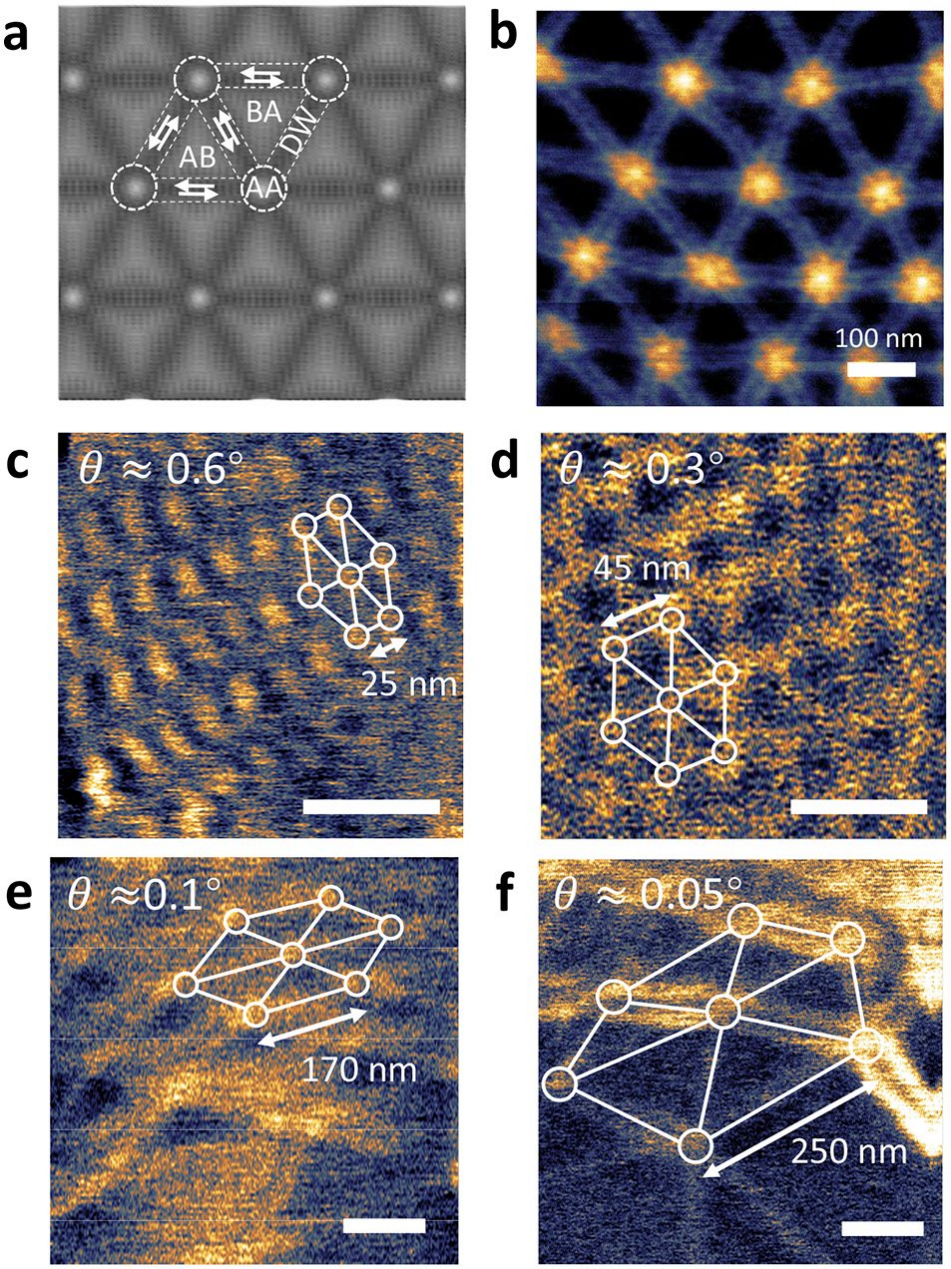
Tuning the moiré period through fine adjustment of the twist angle. (**a**) Schematic of twisted bilayer graphene after atomic reconstruction. (**b**) Near-field infrared image of a typical twist bilayer graphene at high doping level. (**c**–**f**) A series of near-field infrared images of a twistable device at slightly different twist angles, showing a systematically increase of the moiré period. This is achieved by fine tuning of the twist angle using AFM tip. Scale bar: 100 nm.

The long-wave moiré pattern of small-angle tBLG can be directly imaged with a SNOM setup. When the twist angle *θ* is smaller than the critical angle of the lattice reconstruction (*θ*_c_ ≈ 1°), lattice reconstruction becomes significant^30^. Figure 4a shows the schematic diagram of crystal lattice of small-twist angle bilayer graphene after the atomic reconstruction (See more details of molecular dynamics simulations in the Supplementary Information). The atoms in the reconstructed crystal form a tessellation of triangular domains that alternate with AB- and BA-stacking, and shear domain walls are formed between the AB and BA domains. The sudden change of the optical conductivity in the domain wall gives rise to the reflection of plasmon polariton that can be detected by a SNOM system. As an example, Fig. 4b shows near-field optical image of a heavily doped tBLG, from which periodically arranged AB and BA domains and domain walls can be clearly seen, in which the applied gate voltage is 50 V, and the corresponding charge density is about 1.5 × 10^12^ cm^−2^. Based on the imaged moiré period, the twist angle can be derived using Eq. (1).

In Fig. 4c–f, we show a series of moiré patterns of a twistable tBLG device. Initially, the moiré period of this device is ~ 25 nm (Fig. 4c), corresponding to a twist angle of ~ 0.6°. Then we purposely reduce the twist angle of the device step by step with the AFM-tip manipulation. After the first manipulation, the moiré period increases to ~ 45 nm, corresponding to a reduced twist angle of ~ 0.3°. A second fine tuning raises the moiré period up to ~ 170 nm and decreases the twist angle to ~ 0.1°, and a third fine tuning further increases the moiré period to ~ 250 nm and decreases the twist angle to ~ 0.05°. In addition, an inhomogeneity in the twist angle can be observed in the small-twist-angle devices, which also commonly appeals in the direct dry-transferred tBLG devices. In Fig. 4f, the shortest and longest periods are 195 nm and 250 nm, respectively, and the corresponding twist angles are 0.07° and 0.05°, from which an inhomogeneity of 0.02° can be extracted. Note that the blurred near-field infrared signal in Fig. 4c–f is due to the low initial doping level of this device. The initial doping may originate from adsorption of polar molecules on the graphene surface. This example demonstrates that the precision of our twist angle tuning can reach 0.1°.

## Discussion

In conclusion, we demonstrate construction of in-situ twistable bilayer graphene, in which the twist angle of the two graphene monolayers can be in-situ tuned continuously in a large range with high precision. This technique should be applicable to other 2D materials, such as, transition metal dichalcogenide (TMD), black phosphorus, and α-MoO_3_. Our findings enable systematic investigation of the twist angle effects in a single device with largely reduce workload. In addition, the developed large-range and precise tuning of twist angle can help to discover critical transition phenomena that depends sensitively on the twist angle. Besides, the demonstrated in-situ tunable moiré superlattices hold potential for exploring the evolution of the correlated phenomena in near magic angle devices.

## Methods

### Sample fabrication

The hBN substrate was mechanically exfoliated on the silica substrate. The hBN gear and graphene were transferred onto the hBN substrate one by one by the dry transfer methods. After each transfer steps, the sample was annealed in the hydrogen plasma at the temperature of 300 °C to get rid of the adhesive residues. The power of the plasma was 30 W and the flow of H_2_ was 35 SCCM with a pressure of about 47 Pa. The distance between our sample and the center of the coil was about 45 cm.

### Infrared nano-imaging

Our home-made scattering-type SNOM setup was based on a commercial AFM (Bruker Innova). A beam of CO_2_ laser of 10.6 μm wavelength was illuminated onto a gold-coated AFM tip, which generated a near-field hot spot at the nanometer-scale tip apex. Such a largely confined hot spot could provide extra momentum and excite plasmons in graphene. The excited plasmons propagated and reflected at the graphene edges. The reflected plasmons modulated the local field at the tip apex and changed the light scattered by the tip an MCT detector placed in the far field recorded the scattering light intensity as the AFM tip scanned over the graphene. In order to suppress background scattering from the cantilever and the sample, the tip vibrated vertically with an amplitude of ~ 50 nm at a frequency of about 100 kHz, and the detector signal was demodulated at a higher order harmonic frequency by a lock-in amplifier (Zurich Instruments, HF2LI).

## Supplementary Information

Supplementary Information.
