# Supplementary material for: In-situ twistable bilayer graphene
Source: Sci Rep. 2022 Jan 7;12:204. doi: 10.1038/s41598-021-04030-z (PMC8741971; doi:10.1038/s41598-021-04030-z)
Supplement: Supplementary file 1 — Supplementary Information. [file 41598_2021_4030_MOESM1_ESM.pdf]

Supplementary Information for

"*In-situ* twistable bilayer graphene"

Cheng Hu[1], Tongyao Wu[1], Xinyue Huang[1], Yulong Dong[1], Jiajun Chen[1], Zhichun Zhang[1], Bosai Lyu[1], Saiqun Ma[1], Kenji Watanabe[2], Takashi Taniguchi[3], Guibai Xie[4], Xiaojun Li[4], Qi Liang[1], Zhiwen Shi[1*]

[1]Key Laboratory of Artificial Structures and Quantum Control (Ministry of Education), Shenyang National Laboratory for Materials Science, School of Physics and Astronomy, Shanghai Jiao Tong University, Shanghai, 200240, China.

[2]Research Center for Functional Materials, National Institute for Materials Science, 1-1 Namiki, Tsukuba 305-0044, Japan.

[3]International Center for Materials Nanoarchitectonics, National Institute for Materials Science, 1-1 Namiki, Tsukuba 305-0044, Japan.

[4]National Key Laboratory of Science and Technology on Space Science, China Academy of Space Technology (Xi'an), Xi'an, China

* To whom correspondence should be addressed. Email: zwshi@sjtu.edu.cn.

Section 1. The fabrication of device structures

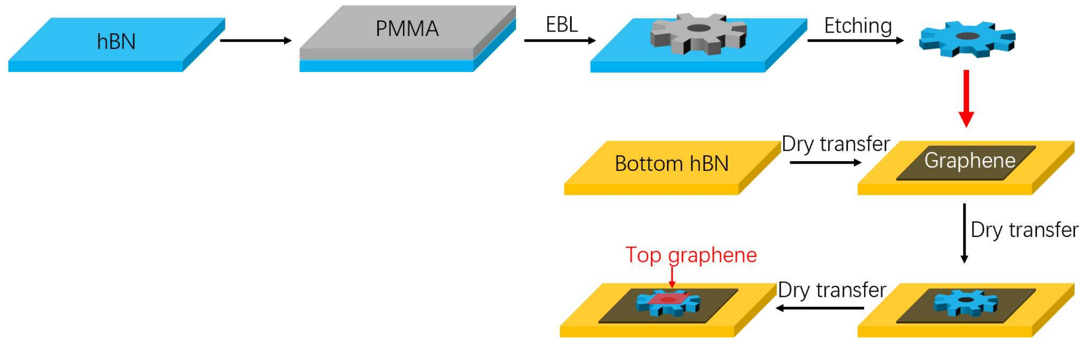

Figure S1. The scheme of steps for the twistable bilayer graphene homostructure device fabrication. The whole steps are on the SiO2/Si substrate.

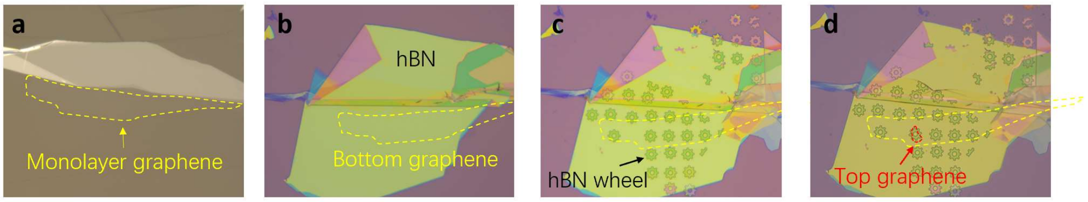

Figure S2. The optical imaging of **a**, The monolayer graphene on PPC. **b,** The monolayer graphene transferred on the hBN substrate as the bottom graphene. **c,** The hBN wheel transferred on the bottom graphene. **d,** The final device after transferred the top graphene on the hBN wheel.

Figure S1 shows the steps for fabricating this graphene homostructure device. The whole processes are on the SiO_2/Si substrate. First, we can fabricate the hBN wheel. The PMMA is spin-coating on the mechanically exfoliated hBN. Then, the electron-beam lithography (EBL) is to pattern the PMMA. After the reactive-ion etching (RIE), with the CHF_3, O_2, we obtain the hBN wheel. On the other hand, the bottom graphene is transferred on the other mechanically exfoliated hBN. After that, the hBN wheel and the top graphene are transferred on the bottom graphene and the hBN wheel step by

step. The bottom graphene on poly propylene carbonate (PPC) and the hBN substrate before and after transformed are shown in Fig. S2a, b, respectively. After removing the residue of the PPC, we transform the hBN gear on the bottom graphene, as shown in Fig. S2c. After removing PPC, we transform the top graphene layer and anneal this device in the final. The optical imaging of the device is shown in Fig. S2d.

Section 2. Raman characteristics of a twistable bilayer graphene device at different twist angles.

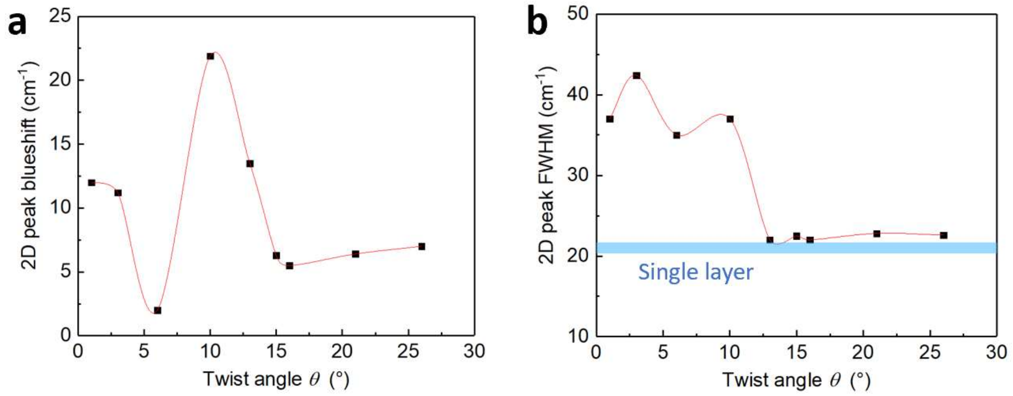

Figure S3. **a**, Relation between the 2D peak shift and the twist angle θ. **b**, Relation between the 2D peak full width at half maximum (FWHM) and the twist angle θ.

Section 3. Molecular dynamics simulation of twisted bilayer graphene before and after structural reconstruction.

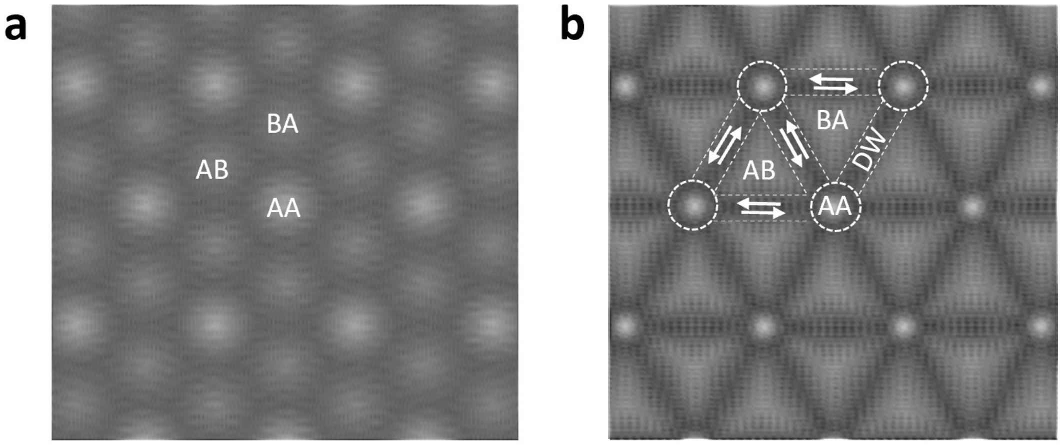

Figure S4. Molecular dynamics simulations of twisted bilayer graphene before (**a**) and after (**b**) structural reconstruction.

The size of the supercells with periodic boundary conditions can be implemented in the molecular dynamics code large-scale atomic/molecular massively parallel simulator (LAMMPS).

The intralayer C–C interactions within each individual layer graphene were computed via the reactive empirical bond order (REBO) force field, and the interlayer interactions between the two graphene layers were described via the recently developed registry-dependent interlayer potential (ILP).

We minimize the total potential energy by relaxing the graphene layer by means of conjugate gradient (CG) and FIRE (a damped dynamics algorithm). When lattice is relaxed, a non-uniform pattern of distortion is found, which is hardly seen when the twist angle is large than 2°. The molecular dynamics simulations of twisted bilayer graphene before and after the atomic reconstruction has been shown in Fig S4.
